# Improvement of germinated brown rice quality with autoclaving treatment

**DOI:** 10.1002/fsn3.1459

**Published:** 2020-02-19

**Authors:** Chuanying Ren, Bin Hong, Xianzhe Zheng, Liqun Wang, Yinglei Zhang, Lijun Guan, Xinmiao Yao, Wengong Huang, Ye Zhou, Shuwen Lu

**Affiliations:** ^1^ Food Processing Research Institute Heilongjiang Academy of Agricultural Sciences Harbin China; ^2^ College of Engineering Northeast Agricultural University Harbin China; ^3^ Institute of Industrial Crops Heilongjiang Academy of Agricultural Sciences Harbin China

**Keywords:** autoclaving, brown rice, gamma‐aminobutyric acid, metabolic syndrome

## Abstract

Germinated brown rice (GBR) is a popular functional food containing considerable amounts of beneficial nutrients and bioactive compounds. Here, autoclaving at 115°C for 20 min was employed to process GBR (AGBR) to evaluate the effect of autoclaving on the nutritional and health function of GBR in microstructure, taste value, aroma, as well as the physiological ingredients. The results showed that autoclaving treatment influenced the starch gelatinization and aroma to improve the taste of cooked AGBR. Autoclaving treatment significantly increased the gamma‐aminobutyric acid (GABA) and ferulic acid levels of AGBR (*p* < .05). In addition, consuming AGBR for 1 month significantly decreased the fasting plasma glucose (FPG), 0.5, 1, and 2 hr postprandial plasma glucose (PPG), triglyceride (TG), total cholesterol (TC), high‐density lipoprotein cholesterol (HDL‐c), and low‐density lipoprotein cholesterol (LDL‐c) in metabolic syndrome (MS) patients (*p* < .05). Therefore, autoclaving treatment, as a promising processing strategy, may both improve the sensory attributes and the nutrition of GBR.

## Graphical abstract

Autoclaving at 115°C for 20 min influenced the starch gelatinization and aroma, and increased the GABA and ferulic acid levels in AGBR. One month eating of AGBR significantly improved the glycolipid metabolism of metabolic syndrome (MS) patients.

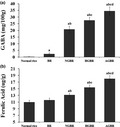

## 1 INTRODUCTION

Rice (*Oryza sativa* L.) is one of the most important cereal grains and is third‐highest in worldwide agricultural production (Gross & Zhao, 2014). The production and consumption of rice are concentrated in Asia, and the production is led by China and India (about 50% of the world production) (Faostat, 2017 2017). Rice is generally consumed wholly by removing the bran layer and germ (Wu, Na, Chen, Jin, & Xu, 2011). However, ignorance of the nutrients enriched in the bran layer, including fiber, iron, calcium, vitamins (B1, B2, E, C, and D), and minerals, is a pity (Patil & Khan, 2011).

Brown rice (BR) is an unmilled whole grain, containing the bran layer and germ. Compared with polished rice, BR is richer in essential nutritional components, such as fiber, iron, calcium, vitamins, and minerals, and in bioactive components, such as gamma‐aminobutyric acid (GABA), ferulic acid, and gamma oryzanol (Matsuo, Sato, Park, Nakamura, & Ohtsuki, 2012; Tomio, Masahiko, Eiko, & Toshiroh, 2002). Previous studies have shown that cooked BR is beneficial for the remission of diverse diseases, such as hypertension, coronary heart disease, diabetes, and metabolic syndrome (MS) (Ravichanthiran et al., 2018; Slavin, 2004). However, BR is less acceptable to consumers due to its inferior sensory quality (Hung, Maeda, & Morita, 2007).

Germination is an effective way to improve sensory quality and the nutrition of BR. Germinated brown rice (GBR) exhibits higher nutrients, sweetness, and digestion and absorption characteristics than that of BR (Wu, Yang, Touré, Jin, & Xu, 2013). Germination improves the taste, nutritional value, and health functions of BR (Fengfeng, Na, Alhassane, Zhengyu, & Xueming, 2013). Germinated brown rice is considered a popular functional food, which may improve the nutrition and health status of people that consume it (Xu, Zhang, Guo, & Qian, 2011). Because the chemical composition of GBR changes dramatically during germination, studies have been performed to optimize germination conditions to maximize the beneficial characteristics of GBR. For example, 3 hr of soaking and 21 hr of gaseous treatment increase the GABA content in GBR compared with conventional soaking (Komatsuzaki et al., 2007). Repeated soaking in tap water at 35°C for 3 hr and incubation at 37°C for 21 hr increased the GABA content in GBR (Thitinunsomboon, Keeratipibul, & Boonsiriwit, 2013). In acidic soaking conditions, exogenous l‐glutamic acid and gibberellin A3 contribute to the accumulation of GABA in japonica GBR (Zhang et al., 2014). Drying is not only an important method for the preservation of GBR, but is also an effective strategy for the improvement of quality. Diverse drying strategies have been applied in the treatment of GBR, such as sun‐drying (Cáceres, Peñas, Martinez‐Villaluenga, Amigo, & Frias, 2017), hot‐air drying (Xu, Zhang, Zhao, Xiong, & Zhang, 2016), microwave drying (Shen et al., 2019; Zheng, Zhu, Lu, Xu, & Sun, 2017), steam drying (Srisang, Varanyanond, Soponronnarit, & Prachayawarakorn, 2011), and fluidized bed drying (Cheevitsopon & Noomhorm, 2011, 2015). Sun‐drying increases γ‐oryzanol, total phenolic compounds, and antioxidant activity in GBR (Cáceres et al., 2017). Hot‐air drying affects the nutritional content, enzymatic hydrolysis, and hardness of GBR (Xu et al., 2016). Microwave drying with ventilation improves the drying uniformity of GBR (Zheng et al., 2017). Superheated steam drying decreases the number of fissured kernels in GBR (Srisang et al., 2011). The superheated steam fluidized bed drying results in a good quality and a high GABA content in parboiled GBR (Cheevitsopon & Noomhorm, 2015). In this study, autoclaving technology was employed to treat GBR (AGBR). The quality of AGBR was evaluated in diverse ways, including the microstructure, taste value, aroma, and nutritional value. The physiological function of AGBR was further evaluated in metabolic syndrome (MS) patients. Our findings revealed a promising processing strategy for GBR, as well as a functional food with high sensory quality and nutrition.

## 2 MATERIAL AND METHODS

### 2.1 BR germination

*Japonica* cultivar, Suijing 18 was provided by the Suihua Branch of Heilongjiang Academy of Agricultural Sciences (Harbin, China). Paddy rice with the husk removed was named as BR. Paddy rice with the husk, bran layer, and germ removed was named as polished rice. Brown rice was washed using clear water three times and germinated in an incubator at 30°C and 95% humidity for 40 hr to produce fresh GBR with a moisture content of 35%–38% (wet basis, w.b.).

### 2.2 GBR treatment

Germinated brown rice was randomly divided into three groups, including natural drying, hot‐air drying, and autoclaving. Natural drying: GBR was spread on a steel plate mesh at a density of 0.8 kg/m^2^, then exposed to sunshine; GBR was stirred every 1 hr until the moisture content was less than 14% (NY/T 3216 criterion, China) (NGBR). Hot‐air drying: GBR was spread on a steel plate mesh at a density of 0.8 kg/m^2^, and exposed to 70°C hot air; GBR was stirred every 10 min until the moisture content was less than 14% (HGBR). Autoclaving treatment: GBR was packed into a high temperature steaming bag (with resistance to 130°C), and sterilized in a high temperature and pressure sterilizing pot at 115°C for 20 min (AGBR).

### 2.3 Rice cooking

Thirty rice samples were washed with clear water and soaked in 40.5 g clear water (weight ratio, 1:1.35) for 30 min. Then, rice was cooked in an electric cooker for 30 min and kept at temperature for 10 min. Samples of cooked rice were randomly selected from the middle layer.

### 2.4 Scanning electron microscope (SEM)

The microstructures of rice and cooked rice were observed by SEM. Simply, the samples were cut into ultrathin slices, fixed in fixative solution for 12 hr, critical point dried, sputter‐coated with gold, and observed under SEM (S‐3400N, Hitachi).

### 2.5 Texture determination

The texture property of cooked rice was detected using a texture analyser (XT. plus). Simply, the samples were placed symmetrically on the objective table. The texture indexes were measured including hardness, adhesiveness, chewiness, gumminess, springiness, cohesiveness, and resilience. The operation parameters were set as a pretest speed of 2 mm/s, post‐test speed of 1.0 mm/s, falling time of 2.0 mm/s, test speed of 1.0 mm/s, and strain of 60% with the test probe SMS P/36R. Each sample was measured six times.

### 2.6 Aroma detection

The rice aroma was detected using gas chromatography–olfactometry–mass spectrometry (GC‐O‐MS). Rice samples were crushed and balanced in a water bath at 60°C for 10 min. Using 50/30 μm divinylbenzene/carboxen/polydimethylsiloxane (Supelco), 50 min of headspace solid‐phase microextraction (HSSPME) was performed, and the solvent phase was desorbed in the gas chromatographic inlet. The volatile flavor compounds were detected by a GC‐MS QP2010GC (Shimadzu) using the following parameters: GC, a GCDB‐WAX column (30 mm × 0.25 mm, 0.25 μm), helium (He) carrier gas, 1.5 ml/min flow rate, 250°C inlet temperature, and 1 min desorption time; MS, electron ionization ion source, 200°C source temperature, 150°C four‐stage temperature, 70 eV electron energy, and 33–400 u scanning mass range. Simultaneously, the aroma was determined by three sensory assessors using an olfactory detector (Gerstel). The volatile flavor compounds were matched with the National Institute of Standards and Technology standard reference database, and the retention index (RI) was determined by chromatographic scanning of C6‐C30 N‐alkanes. The odor activity value (OAV) was determined using an internal and external standard method.

### 2.7 GABA measurement

GABA content in rice was measured using high‐performance liquid chromatography (HPLC) in accordance with the criteria of NY/T 2890‐2016 (Agricultural Industry Standards of China). HPLC was performed on an LCMS‐8050 (Shimadzu) using the following parameters: a C18 column (3 µm diameter, 250 mm × 4.6 mm), acetonitrile‐sodium acetate trihydrate (35:65) mobile phase, 1.0 ml/min flow rate, 30°C column temperature, and 436 nm detection wavelength.

### 2.8 Ferulic acid measurement

Ferulic acid content in rice was measured by HPLC in accordance with a previous study (Wang, Min, Cui, & Hou, 2010). HPLC was performed on an LCMS‐8050 (Shimadzu) using the following parameters: a Diamonsil column (5 µm diameter, 250 mm × 4.6 mm), methanol‐1% acetic acid (50:50) mobile phase, 1.0 ml/min flow rate, 25°C column temperature, and 320 nm detection wavelength.

### 2.9 MS patients

A total of 112 MS patients (62 males, 50 females; 18–70 years old) were screened from the General Hospital of the Chinese People's Liberation Army (Beijing, China) between February 2017 and October 2018. These patients met three or more than three of the following criteria: (a) abdominal obesity, male waistline > 90 cm and female waistline > 85 cm; (b) body mass index ≥ 22 kg/m^2^; (c) dyslipidemia, triglyceride (TG) ≥ 1.7 mmol/L and high‐density lipoprotein cholesterol (HDL‐c) < 1.04 mmol/L; (d) hypertension, systolic/diastolic blood pressure ≥ 130/85 mm Hg; (e) hyperglycemia, fasting plasma glucose (FPG) ≥ 6.1 mmol/L and/or 2 hr postprandial plasma glucose (PPG) ≥ 7.8 mmol/L; (f) diabetes history. Patients who had severe liver, kidney, and stomach diseases were excluded from this study. This study was approved by the local Institutional Review Board, and informed consent was obtained from all patients.

### 2.10 Rice intake

MS patients were randomly divided into four groups, including polished rice, BR, HGBR, and AGBR (*N* = 28 each group). A standard meal was eaten by these patients for 1 month, which included 30 g sauced beef, 150 g salad, 50 g eggs, and 100 rice (50 g sample rice + 50 g polished rice). No coarse cereals were eaten during the experimental period.

### 2.11 Blood sugar and lipid measurement

Glucose metabolism parameters, including FPG, 0.5, 1, and 2 hr PPG, as well as lipid metabolism parameters, including TG, total cholesterol (TC), HDL‐c, and low‐density lipoprotein cholesterol (LDL‐c), were measured using an automatic biochemical analyser (Cobas8000, Roche) before and after 1 month of treatment.

### 2.12 Statistical analyses

Data are expressed as the mean ± standard deviation (*SD*). Statistical analysis was performed by SPSS version 17.0 (SPSS Inc., Chicago, IL). Comparison between different groups was determined by *t* test (two groups) or one‐way ANOVA (more than two groups). A *p*‐value less than .05 was considered significantly different.

## 3 RESULTS

### 3.1 Microstructures of GBR

**Figure 1:**
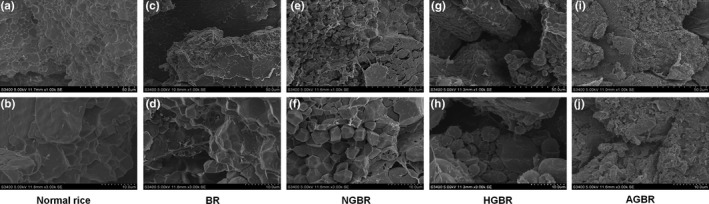
The microstructure of rice was observed by scanning electron microscope (SEM). a, c, e, g, i (1,000×); b, d, f, h, j (3,000×). AGBR, autoclaving‐treated germinated brown rice; BR, brown rice; HGBR, hot‐air drying‐treated germinated brown rice; NGBR, natural drying‐treated germinated brown rice

The rice microstructures were observed by SEM. As shown in Figure 1a,b, the cross‐section of polished rice presents a complete reticular structure accompanied with a smooth surface and few cracks (Figure 1a,b). Similar microstructures were observed in brown rice (Figure 1c,d). After germination, a rough cross‐section, massive cracks, as well as obvious starch granule‐like structures were observed in NGBR (Figure 1e,f). Obvious starch gelatinization was observed in AGBR, which presented an irregular particle surface and severe adhesion (Figure 1i,j). The starch gelatinization degree of HGBR was between that of NGBR and AGBR (Figure 1g,h).

### 3.2 Microstructures of cooked GBR

**Figure 2:**
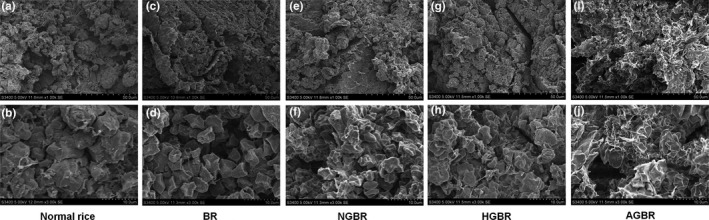
The microstructure of cooked rice was observed by scanning electron microscope (SEM). a, c, e, g, i (1,000×); b, d, f, h, j (3,000×). AGBR, autoclaving‐treated germinated brown rice; BR, brown rice; HGBR, hot‐air drying‐treated germinated brown rice; NGBR, natural drying‐treated germinated brown rice

As shown in Figure 2a,b, serious gelatinization of starch granules is observed in cooked polished rice, exhibiting obvious adhesion (Figure 1a,b). Weak gelatinization of starch granules was observed in cooked BR, exhibiting a granular sensation without adhesion (Figure 1c,d). After germination, the starch granules in cooked NGBR and HGBR were irregular and small in shape with adhesiveness (Figure 1e‐h). Since autoclaving ripened AGBR before cooking, to some degree, the gelatinization of starch granules was the most serious in the cooked AGBR among the five kinds of cooked rice, exhibiting serious adhesion and broken granules (Figure 1i,j).

### 3.3 Autoclaving improved the taste value of cooked BR

The taste value of cooked rice was detected using a texture analyser. As shown in Table 1, cooked BR exhibits the highest hardness, chewiness and gumminess, and the lowest adhesiveness, springiness, cohesiveness, and resilience among the five kinds of cooked rice (*p* < .05). After germination, the hardness, chewiness, and gumminess were significantly decreased, and the adhesiveness, springiness, cohesiveness, and resilience were significantly increased in cooked NGBR and HGBR (*p* < .05). Except for springiness, the taste value was better in cooked HGBR than in cooked NGBR (*p* < .05). Notably worthily, the taste value of cooked AGBR was greatly improved by autoclaving. The hardness, adhesiveness, chewiness, and resilience of cooked AGBR were only second to cooked polished rice (*p* < .05). The gumminess, springiness, and cohesiveness of cooked AGBR were not significantly different with that of cooked polished rice (Table 1).

**Table 1:** The taste value of cooked rice

Parameters	Hardness (g)	Adhesiveness (g/s)	Chewiness (g)	Gumminess	Springiness	Cohesiveness	Resilience
Polished rice	530.57 ± 69.12	−10.94 ± 2.45	244.30 ± 21.45	318.85 ± 32.58	0.76 ± 0.05	0.600 ± 0.09	0.330 ± 0.02
BR	1,376.40 ± 178.92^a^	−1.17 ± 0.16^a^	377.79 ± 39.78^a^	590.80 ± 63.49^a^	0.58 ± 0.04^a^	0.408 ± 0.05^a^	0.227 ± 0.03^a^
NGBR	1,099.85 ± 165.42^ab^	−1.68 ± 0.28^ab^	334.48 ± 26.18^ab^	482.46 ± 36.73^ab^	0.68 ± 0.06^ab^	0.412 ± 0.03^ab^	0.286 ± 0.02^ab^
HGBR	813.09 ± 109.14^abc^	−2.58 ± 0.27^abc^	317.37 ± 23.47^abc^	418.74 ± 34.21^abc^	0.67 ± 0.06^ab^	0.435 ± 0.03^abc^	0.305 ± 0.02^abc^
AGBR	641.37 ± 77.35^abcd^	−2.92 ± 0.31^abcd^	267.53 ± 31.69^abcd^	317.54 ± 24.88^bcd^	0.74 ± 0.07^bcd^	0.596 ± 0.06^bcd^	0.320 ± 0.02^abcd^

Abbreviations: AGBR, autoclaving‐treated germinated brown rice; BR, brown rice; HGBR, hot‐air drying‐treated germinated brown rice; NGBR, natural drying‐treated germinated brown rice.

a, *p* < .05 versus Polished rice; b, *p* < .05 versus BR; c, *p* < .05 versus NGBR; d, *p* < .05 versus HGBR.

### 3.4 Autoclaving changed the aroma of GBR

The rice aroma was detected by GC‐O‐MS. The volatile flavor compounds of polished rice mainly included styrene, benzaldehyde, (E)‐2‐octenol, nonanal, naphthalene, (E)‐2‐decenoaldehyde, 1‐methylnaphthalene, and 2‐methoxy‐4‐vinylphenol, presenting the aroma of flower, almond, potato, citrus, mint, fat, and cloves. The volatile flavor compounds in BR were less diverse than in polished rice, which were dominated by styrene, (E)‐2‐octenol, naphthalene, and (E)‐2‐decenoaldehyde. Germination decreased levels of styrene, eliminated the naphthalene, and (E)‐2‐decenoaldehyde, and enriched some other compounds, such as hexanal, benzaldehyde, 1‐octen‐3‐ol, and nonanal in NGBR. Except for the elimination of 1‐octen‐3‐ol and (E)‐2‐octenol, HGBR exhibited similar volatile flavor compounds as NGBR, and presented the aroma of fruit, flower, almond, and citrus. Compared with NGBR, the autoclaving treatment increased the levels of styrene, 1‐octen‐3‐ol, and (E)‐2‐octenol, and enriched levels of 2,3‐dihydrobenzofuran, (E)‐2‐decenoaldehyde, and 1‐methylnaphthalene in AGBR (Table 2).

**Table 2:** The volatile flavor compounds in rice

Compounds	RI	Content (μg/kg) (OVA)					
		AGBR	HGBR	NGBR	BR	Polished rice	Aroma
Hexanal	800	0.96 (0.21)	39.05 (6.46)	41.05 (9.12)	—	—	Fruit
Styrene	893	275.41 (0.38)	229.87 (0.28)	241.41 (0.33)	381.21 (0.52)	606.93 (0.83)	Flower
Benzaldehyde	962	—	28.18 (0.06)	33.67 (0.08)	—	64.63 (0.18)	Almond
1‐octen‐3‐ol	980	53.59 (53.59)	—	18.47 (18.47)	—	—	Mushroom
(E)‐2‐Octenol	1,067	47.31	—	13.51	15.07	107.28	Potato
Nonanal	1,104	—	314.50 (314.50)	347.52 (347.52)	—	116.26 (116.26)	Citrus
Naphthalene	1,182	—	—	—	9.95 (0.71)	21.49 (1.54)	Mint
2,3‐Dihydrobenzofuran	1,224	35.36	—	—	—	8.78	—
(E)‐2‐decenoaldehyde	—	14.42 (48.07)	—	—	12.55 (41.83)	155.38 (517.93)	Fat
1‐Methylnaphthalene	1,307	22.00 (1.57)	—	—	—	13.05 (0.93)	Mint
2‐Methoxy‐4‐vinylphenol	1,317	—	—	—	—	8.25 (2.75)	Clove
Lauric aldehyde	1,409	—	—	—	4.28	68.51	—
Geranyl acetone	1,453	—	—	—	9.10	26.34	—
2,6‐di‐tert‐butyl‐4‐methylphenol	1,513	—	418.47	416.55	16.36	89.01	—
2,2,4‐trimethylpentanediol isobutyl ester	1,588	—	16.34	22.34	—	34.38	—
Di‐tert‐dodecyl disulfide	—	16.20	—	—	4.88	—	—
Tertiary hexadecathiol	—	12.07	3.73	4.89	5.68	113.45	—

Abbreviations: AGBR, autoclaving‐treated germinated brown rice; BR, brown rice; HGBR, hot‐air drying‐treated germinated brown rice; NGBR, natural drying‐treated germinated brown rice; OAV, odor activity value; RI, retention index.

### 3.5 Autoclaving improved the nutritional value of GBR

**Figure 3:**
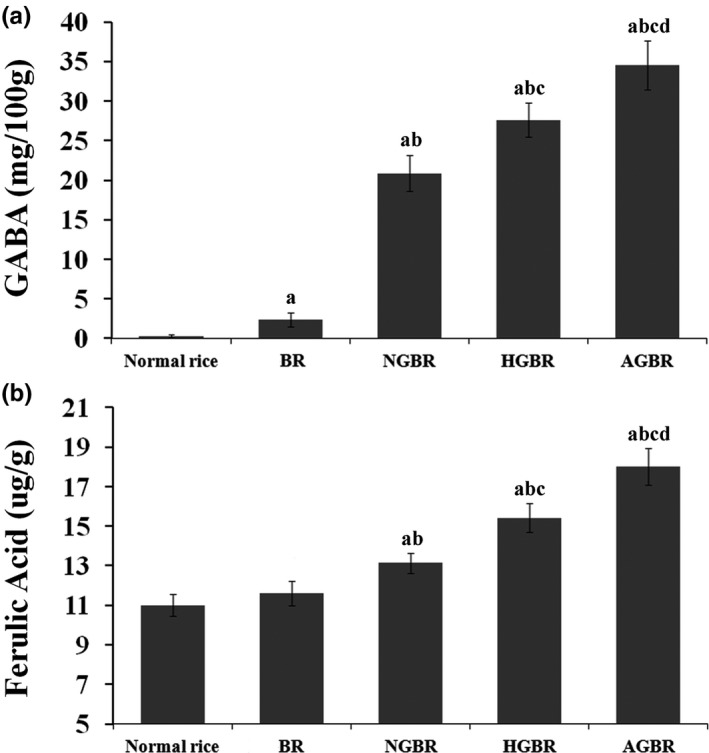
The gamma‐aminobutyric acid (GABA) and ferulic acid levels in rice. AGBR, autoclaving‐treated germinated brown rice; BR, brown rice; HGBR, hot‐air drying‐treated germinated brown rice; NGBR, natural drying‐treated germinated brown rice. a, *p* < .05 versus Polished rice; b, *p* < .05 versus BR; c, *p* < .05 versus NGBR; d, *p* < .05 versus HGBR

The nutritional value of rice was mainly evaluated by the levels of GABA and ferulic acid. As shown in Figure 3a, GABA levels are significantly higher in BR than in polished rice (*p* < .05). No significant difference was revealed in the ferulic acid levels between BR and polished rice (Figure 3b). Germination significantly increased the GABA and ferulic acid levels in NGBR (*p* < .05). Hot‐air treatment further increased the GABA and ferulic acid levels in HGBR (*p* < .05). Levels of GABA and ferulic acid were the highest in AGBR among the five kinds of rice (*p* < .05) (Figure 3a,b).

### 3.6 GBR improved the glycolipid metabolism of MS patients

The effect of GBR on glycolipid metabolism was evaluated in MS patients. As shown in Table 1, eating HGBR for 1 month significantly decreased the FPG, as well as 0.5, 1, and 2 hr PPG in MS patients (*p* < .05). Eating HGBR for 1 month also significantly decreased the TG, TC, as well as HDL‐c and LDL‐c in MS patients (*p* < .05). AGBR exhibited a more obvious hypolipidemic and hypoglycemic effect on MS patients than that of HGBR. However, the glycolipid metabolism of MS patients was not significantly influenced by either polished rice or BR (Tables 3 and 4).

**Table 3:** Glucose metabolism in patients with metabolic syndrome (MS)

Parameters	FPG (mmol/L)		0.5 hr PPG (mmol/L)		1 hr PPG (mmol/L)		2 hr PPG (mmol/L)	
	Before	One month later	Before	One month later	Before	One month later	Before	One month later
Polished rice	7.82 ± 1.57	7.79 ± 1.36	11.79 ± 1.35	12.13 ± 1.40	13.27 ± 1.27	12.96 ± 1.19	9.57 ± 0.96	9.16 ± 1.06
BR	7.40 ± 1.34	7.09 ± 1.09	11.35 ± 1.27	11.78 ± 1.38	12.84 ± 1.66	13.17 ± 1.37	9.93 ± 1.09	9.64 ± 1.34
HGBR	7.60 ± 1.42	6.01 ± 1.13*	11.95 ± 1.46	9.83 ± 0.99*	13.03 ± 1.24	10.38 ± 1.32*	9.82 ± 1.61	7.81 ± 1.32*
AGBR	7.49 ± 1.59	5.63 ± 1.22*	11.87 ± 1.65	8.16 ± 1.33*	12.74 ± 1.43	9.66 ± 1.29*	8.92 ± 3.22	7.08 ± 4.03*

Abbreviations: AGBR, autoclaving‐treated germinated brown rice; BR, brown rice; FPG, fasting plasma glucose; HGBR, hot‐air drying‐treated germinated brown rice; PPG, postprandial plasma glucose.

* *p* < .05 versus Before intake (Before).

**Table 4:** Lipid metabolism in patients with metabolic syndrome (MS)

Parameters	TG (mmol/L)		TC (mmol/L)		HDL‐c (mmol/L)		LDL‐c (mmol/L)	
	Before	One month later	Before	One month later	Before	One month later	Before	One month later
Polished rice	3.08 ± 0.62	2.95 ± 0.56	5.19 ± 1.35	5.13 ± 1.40	1.27 ± 0.12	1.31 ± 0.11	3.17 ± 0.96	3.22 ± 0.79
BR	2.93 ± 0.72	3.16 ± 0.89	5.17 ± 1.15	5.21 ± 1.09	1.29 ± 0.13	1.25 ± 0.12	3.22 ± 0.66	3.16 ± 1.02
HGBR	2.81 ± 0.64	1.80 ± 0.76*	5.14 ± 1.28	4.35 ± 1.13*	1.30 ± 0.12	1.14 ± 0.09*	3.24 ± 0.86	2.85 ± 0.67*
AGBR	2.74 ± 0.60	1.61 ± 0.82*	5.16 ± 1.02	3.97 ± 1.08*	1.27 ± 0.11	0.98 ± 0.12*	3.04 ± 0.65	2.41 ± 0.71*

Abbreviations: AGBR, autoclaving‐treated germinated brown rice; BR, brown rice; HDL‐c, high‐density lipoprotein cholesterol; HGBR, hot‐air drying‐treated germinated brown rice; LDL‐c, low‐density lipoprotein cholesterol; TC, total cholesterol; TG, triglyceride.

* *p* < .05 versus Before intake (Before).

## 4 DISCUSSION

Brown rice is a whole grain containing large amounts of beneficial nutrients and bioactive compounds. Because germination improves the beneficial effects of BR, GBR has been widely served as a functional food (Cáceres, Martínez‐Villaluenga, Amigo, & Frias, 2014). Germination can influence the characteristics of BR in diverse ways, such as the texture, sensory quality, and nutrition. In this study, we first evaluated the texture of GBR using SEM and a texture assay. We found that germination induced the formation of a rough cross‐section, cracks, and starch granule‐like structures in NGBR, and irregular, small, and adhesive starch granules in cooked NGBR. Germination decreased the hardness, chewiness, and gumminess, and increased the adhesiveness, springiness, cohesiveness, and resilience in cooked NGBR. These findings indicated that germination improved the taste value of cooked BR. In addition, we also found that the texture value, except for springiness, was better in cooked HGBR than in cooked NGBR. This phenomenon may be explained by the hypothesis that heating ripened GBR before cooking, to some degree. Notably, cooked AGBR exhibited a greatly improved taste value, which was similar to cooked polished rice. This improvement may be attributed to the serious starch gelatinization induced by autoclaving.

Aroma is an important quality index of rice. In this study, the rice aroma of GBR was analyzed by GC‐O‐MS. We found that the volatile flavor compounds in BR were dominated by styrene, (E)‐2‐octenol, naphthalene, and (E)‐2‐decenoaldehyde, which were less diverse than in polished rice. This phenomenon may be explained by the hypothesis that the presence of the bran layer blocks the volatilization of some flavor compounds in BR. The specific aroma of BR, which is different from polished rice is mainly manifested in the intrinsic compounds within the bran layer and germ (Xia, Mei, Yu, & Li, 2017). In addition, we found that germination decreased levels of styrene, and eliminated naphthalene, and (E)‐2‐decenoaldehyde in GBR. These volatile flavoring components may be decreased by the steeping and metabolic reactions that occur during germination. In contrast to NGBR, 1‐octen‐3‐ol and (E)‐2‐octenol were not observed in HGBR, indicating these two components were thermolabile. A previous study showed that high hydrostatic pressure enhances the flavor components of GBR, particularly aldehydes, ketones, and alcohols (Xia et al., 2017). Here, autoclaving treatment increased the levels of styrene, 1‐octen‐3‐ol, and (E)‐2‐octenol, and enriched levels of 2,3‐dihydrobenzofuran, (E)‐2‐decenoaldehyde, and 1‐methylnaphthalene in AGBR. Our findings indicated that the autoclaving treatment improved the aroma of GBR through the enrichment of some flavor compounds.

GABA is an important inhibitory neurotransmitter in the brain that exists naturally in foods. GABA exhibits many health benefits in humans, such as reduction of blood pressure and cholesterol, improvement of kidney and liver activity, and inhibition of tumors (Diana, Quílez, & Rafecas, 2014). In rice, GABA is mainly distributed in the bran layer and germ. Previous studies have shown that germination can obviously increase the GABA content in GBR through the activation of glutamic acid decarboxylases, amylases, and proteases (Khwanchai, Chinprahast, Pichyangkura, & Chaiwanichsiri, 2014; Lu, Goto, & Nishizu, 2010; Ng, Huang, Chen, & Su, 2013). Consistent with previous studies, we found that germination significantly increased the GABA content in GBR. Notably, hot‐air treatment and autoclaving further increased levels of GABA in GBR, and the improvement was stronger after autoclaving than the hot‐air treatment. These results indicated that GABA was an active thermostable component, and thermal stress promoted the accumulation of GABA in GBR. Ferulic acid is known as a potent antioxidant of scavenging free radicals that exist widely in fruits and vegetables (Zhou et al., 2018). Previous studies have shown that ferulic acid is richer in GBR than in BR and is the most abundant phenolic compound in GBR (Ohtsubo, Suzuki, Yasui, & Kasumi, 2005; Su, Kozo, & Hiroshi, 2004). In this study, the ferulic acid results were consistent with the GABA results. Our findings indicated that thermal stress, especially autoclaving could improve the nutritional value of GBR by increasing the levels of GABA and ferulic acid.

MS is a group of risk factors that is accompanied by a high risk of cardiovascular disease and diabetes (Grundy, 2006). Germinated brown rice exhibits great therapeutic potential for MS‐induced diseases. For example, GBR ameliorates the risk of cardiovascular disease by reducing weight gain and improving lipid metabolism in hypercholesterolemic rats (Imam et al., 2014). Germinated brown rice suppresses body weight gain, improves lipid profiles, and reduces leptin levels and white adipose tissue mass in obese rats with a high‐fat diet (Lim, Yong, Mohtarrudin, & Su, 2016). To identify the function of GBR in MS, the glycolipid metabolism of MS patients receiving GBR diet was analyzed. We found that eating HGBR and AGBR for 1 month could significantly decrease the FPG, PPG (0.5, 1, and 2 hr), TG, TC, HDL‐c, and LDL‐c in MS patients. These results indicated that GBR was beneficial to the improvement of glycolipid metabolism in MS patient. Our findings are consistent with previous studies. It has been reported that a GBR diet favorably improves the blood concentrations of FPG, fructosamine, TG, and TC in patients with impaired fasting glucose or type 2 diabetes (Hsu et al., 2008). A GBR diet is useful in controlling body weight, blood pressure, as well as glucose and lipid levels in women with impaired glucose tolerance (Bui et al., 2014). It is noteworthy that AGBR exhibited more obvious hypolipidemic and hypoglycemic effects than that of HGBR in MS patients. This phenomenon may be partially attributable to the autoclaving‐induced high levels of GABA in AGBR.

In conclusion, autoclaving treatment improved the taste value, changed the aroma, and increased GABA and ferulic acid levels in GBR. An AGBR diet improved the glycolipid metabolism of MS patients. Thus, AGBR may be a promising functional food with a high sensory quality and nutritional value.

## CONFLICT OF INTEREST

Authors declare that they have no conflict of interest.

## ETHICAL APPROVAL

This study was approved by the Institutional Review Board of Chinese PLA General Hospital (Beijing, China) and conformed to the declaration of Helsinki, USA. Written informed consent was obtained from all participants.
